# Regulation of miRNA Expression by Low-Level Laser Therapy (LLLT) and Photodynamic Therapy (PDT)

**DOI:** 10.3390/ijms140713542

**Published:** 2013-06-27

**Authors:** Toshihiro Kushibiki, Takeshi Hirasawa, Shinpei Okawa, Miya Ishihara

**Affiliations:** Department of Medical Engineering, National Defense Medical College 3-2 Namiki, Tokorozawa, Saitama 359-8513, Japan

**Keywords:** low-level laser therapy (LLLT), phototherapy, photodynamic therapy (PDT), miRNA

## Abstract

Applications of laser therapy, including low-level laser therapy (LLLT), phototherapy and photodynamic therapy (PDT), have been proven to be beneficial and relatively less invasive therapeutic modalities for numerous diseases and disease conditions. Using specific types of laser irradiation, specific cellular activities can be induced. Because multiple cellular signaling cascades are simultaneously activated in cells exposed to lasers, understanding the molecular responses within cells will aid in the development of laser therapies. In order to understand in detail the molecular mechanisms of LLLT and PDT-related responses, it will be useful to characterize the specific expression of miRNAs and proteins. Such analyses will provide an important source for new applications of laser therapy, as well as for the development of individualized treatments. Although several miRNAs should be up- or down-regulated upon stimulation by LLLT, phototherapy and PDT, very few published studies address the effect of laser therapy on miRNA expression. In this review, we focus on LLLT, phototherapy and PDT as representative laser therapies and discuss the effects of these therapies on miRNA expression.

## 1. Low-Level Laser Therapy (LLLT) and Its Effects on miRNA Expression

A laser (light amplification by stimulated emission of radiation) is a device that generates electromagnetic radiation that is relatively uniform in wavelength, phase and polarization. This technology was originally described by Maiman in 1960 in the form of a ruby laser [1]. The properties of lasers have allowed for numerous medical applications, including their use in surgery, activation of photodynamic agents and various ablative therapies in cosmetics, all of which are based on heat generated by the laser beam, in some cases, leading to tissue destruction [2–9]. These applications of lasers are considered “high-energy”, because of their intensities, which range from about 1–100 watt (W)/cm^2^.

This paper will address another type of laser application, low-level laser therapy (LLLT), which elicits its effects through non-thermal means. This field was initiated by the work of Mester *et al*., who in 1967 reported non-thermal effects of lasers on mouse hair growth [10]. In a subsequent study, the same group reported acceleration of wound healing and improvement in the post-wounding regeneration ability of muscle fibers using a 1 J/cm^2^ ruby laser [11]. Since those early days, numerous *in vitro* and *in vivo* studies of LLLT in the context of regenerative medicine have demonstrated a wide variety of therapeutic effects, including reduction of pain, anti-inflammatory effects and wound healing. According to da Silva *et al*. [12], the types of laser most frequently used for wound healing and tissue repair are helium neon (He-Ne) lasers and diode lasers, including gallium-aluminum-arsenic (Ga-Al-As), arsenic-gallium (As-Ga) and indium-gallium-aluminum-phosphide (In-Ga-Al-P) lasers.

One of the most distinctive features of LLLT relative to other modalities is that the effects are mediated not through induction of thermal effects, but rather, through a process, still not clearly defined, called “photobiostimulation”. Because this effect of LLLT apparently does not depend on coherence, it is therefore possible to achieve photobiostimulation using non-laser light-generating devices, such as inexpensive light-emitting diode (LED) technology [13–17]. To date, several mechanisms of biological action have been proposed, although none have been clearly established. These include augmentation of cellular ATP levels [18–20], manipulation of inducible nitric oxide synthase (iNOS) activity [21–25], suppression of inflammatory cytokines, such as TNF-alpha [19,26–28], IL-1beta [28–30], IL-6 [28,31–34] and IL-8 [28,31,32,35], upregulation of growth factors, such as PDGF, IGF-1, NGF and FGF-2 [30,36–38], alteration of mitochondrial membrane potential [39–42], due to chromophores found in the mitochondrial respiratory chain [43–45], stimulation of protein kinase C (PKC) activation [46], manipulation of NF-kappaB activation [47], induction of reactive oxygen species (ROS) [48,49], modification of extracellular matrix components [50], inhibition of apoptosis [39], stimulation of mast cell degranulation [51] and upregulation of heat shock proteins [52]. We have also proposed that LLLT influences cell differentiation following laser stimulation [53–55].

Unfortunately, these effects have been demonstrated using a variety of laser devices in non-comparable models. To add to the confusion, dose-dependency seems to be confined to a very narrow range, and in numerous systems, the therapeutic effects disappear with increased dose. Consequently, only two studies of miRNA expression dynamics following LLLT have been reported to date, by Wang *et al*. [56] and Gu *et al*. [57]. With the exception of those studies, no data are currently available regarding the overall changes in the global expression of many hundreds of miRNAs following LLLT. Wang *et al*. [56] showed that LLLT increases the migration, proliferation and viability of rat mesenchymal stem cells (MSCs) and, also, activates the expression of various miRNAs. Using a diode laser (wavelength: 635 nm, 0.5 J/cm^2^), they found that the proliferation rate and expression of cell cycle-associated genes increased in a time-dependent manner following LLLT treatment of MSCs. Microarray assays revealed subsets of miRNAs that were regulated by LLLT: 19 miRNAs were upregulated and 15 miRNAs were downregulated (Table 1); these dynamic changes were confirmed by quantitative real-time PCR.

The most highly upregulated miRNA was miR-193. Gain- and loss-of-function experiments demonstrated that miR-193 levels regulate the proliferation of MSCs of both humans and rats; in particular, blockade of miR-193 repressed the MSCs proliferation induced by LLLT. However, this miRNA apparently does not affect apoptosis or differentiation. In addition, Wang *et al*. found that miR-193 regulated expression of cyclin-dependent kinase 2 (CDK2). Bioinformatic analyses and luciferase reporter assays revealed that inhibitor of growth family, member 5 (ING5), was the most likely target of miR-193 to functionally regulate proliferation and CDK2 expression; indeed, the mRNA and protein levels of ING5 are regulated by miR-193. Furthermore, inhibition of ING5 by small interfering RNA (siRNA) upregulated both MSC proliferation and the expression of CDK2. Another miRNA, miR-335, has been shown by others to regulate the proliferation and migration of MSCs [58], so it is likely to play an important role in MSC proliferation after LLLT. Moreover, several studies have shown that LLLT also stimulates cell differentiation [53–55,59–75], and future work should reveal miRNAs specifically involved in mediating this effect.

Although some literature reported that tumor or apoptosis related miRNAs were induced by UV irradiation to cells [76–81], Gu *et al*. reported UV-phototherapy and its effect on miRNA expression [57]. They showed the effect of narrow-band ultraviolet B (NB-UVB) irradiation on miR-21 and -125b expression in psoriatic epidermis. Psoriasis is an inflammatory skin disease in which dysregulation of p63, a member of the p53 family that is crucial for skin development and maintenance, has been demonstrated [82–84]. Involvement of miR-203, miR-21 and miR-125b were implicated in the regulation of p63 or p53 in the pathogenesis of psoriasis. Skin biopsies from 12 psoriasis patients were collected before, during and after NB-UVB therapy. The p63 expression was not significantly affected, whereas NB-UVB phototherapy significantly decreased expression of miR-21and increased miR-125b levels. Since NB-UVB phototherapy is commonly used in the treatment of psoriasis [85–87], those results indicate a complex mechanism of p63 regulation, which merits further investigation in order to achieve better long-term clinical improvement.

## 2. Photodynamic Therapy and Its Effects on miRNA Expression

Photodynamic therapy (PDT), a class of laser therapy, is a photochemical modality approved for the treatment of various cancers and diseases in which neovascularization occurs [88,89]. The PDT process consists of injecting a photosensitizer, which selectively accumulates at the lesion site, followed by local irradiation of the tumor with light of an appropriate wavelength to activate a specific drug [90]. Irradiation leads to the generation of singlet oxygen and other reactive oxygen species (ROS) [91]. PDT is being considered not only as palliative therapy, but also as a treatment option for early-stage skin, lung, cervical and esophageal cancers, as well as basal-cell carcinomas. Currently, PDT has been approved for localized diseases and precancerous lesions, such as bladder cancers, pituitary tumors and glioblastomas [92,93]. Furthermore, numerous ongoing clinical studies have been designed to optimize the conditions for PDT; subsequently, PDT has been approved in several countries.

Upon absorption of one or more photons, the excited photosensitizer undergoes one of two possible reactions (type I or/and II) with a neighboring oxygen molecule, yielding ROS [94]. These ROS oxidize various cellular substrates, affecting cellular functions and resulting in cell death. The ROS that are produced during PDT destroy tumors by multiple mechanisms: in contrast to most conventional cytotoxic agents, which usually only trigger apoptotic cell death, PDT can cause cell death by necrosis and/or apoptosis.

**Figure 1:**
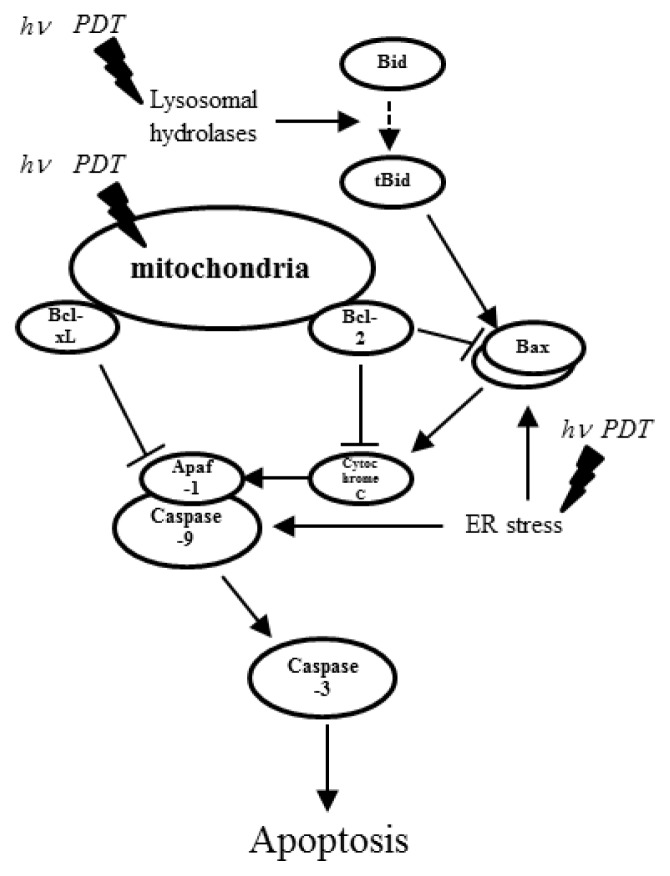
Representative signaling pathways of apoptosis induced by photodynamic therapy (PDT). Depending on the nature of the photosensitizer and its intracellular localization, the initial photodamage can involve different molecules, with the consequent activation of specific death pathways that converge on mitochondria. Mitochondria-localized photosensitizer can cause immediate and light-dependent photodamage to the anti-apoptotic Bcl-2 and Bcl-xL proteins, prompting the release of caspase-activating molecules. Lysosomal hydrolases and ER stress also induce Bax-mediated caspase activation.

The direct destruction of cancer cells (necrosis) by PDT is caused by irreversible damage to the plasma membrane and intracellular organelles, including the mitochondria, lysosomes, Golgi apparatus and endoplasmic reticulum (ER). The mechanisms of PDT-induced apoptosis have been described by many studies. Apoptosis, or programmed cell death, is one mechanism that mediates toxicity in the target tissue following PDT [95]. Apoptosis involves a cascade of molecular events leading to orderly cellular death without an inflammatory response [96–98]. The initiation of apoptosis involves a complex network of signaling pathways, both intrinsic and extrinsic to the individual cell, which are regulated, in part, by pro- and anti-apoptotic factors [96]. The initial damage can involve different molecules, ultimately leading to activation of specific death pathways. Mitochondria-localized photosensitizers can cause immediate and light-dependent photodamage to mitochondrial components, such as the anti-apoptotic Bcl-2, Bcl-xL and the other apoptosis-related proteins, prompting the release of caspase-activating molecules [99]. Photosensitizers that accumulate in the lysosomes or mitochondria and which were excited by laser light can induce Bax-mediated caspase activation (Figure 1).

Another important cellular factor induced by PDT and released from necrotic tumor cells is heat-shock protein 70 (Hsp70) [100]. Hsp70 is significantly induced after stress; when it remains within the cell, it chaperones unfolded proteins and prevents cell death by inhibiting the aggregation of cellular proteins. Hsp70 directly binds to the caspase-recruitment domain of apoptotic-protease activating factor 1 (Apaf-1), thereby preventing the recruitment of Apaf-1 oligomerization and association of Apaf-1 with procaspase 9. These properties not only enable intracellular Hsp70 to inhibit cancer-cell death by apoptosis, but also promote the formation of stable complexes with cytoplasmic tumor antigens. These antigens can then either be expressed at the cell surface or escape intact from dying necrotic cells to interact with antigen-presenting cells, thereby stimulating an anti-tumor immune response.

The mechanisms of cell death following PDT have been thoroughly summarized in the literature [95,101–104]. A better understanding of the molecular differences between apoptosis and necrosis and identification of the crosstalk between these programs will certainly be crucial to the development of new PDT modalities aimed at increasing the efficiency of cancer-cell killing.

Another inherent consequence of PDT is local hypoxia, which can arise either directly, from oxygen consumption during treatment [105–107], or indirectly, from the destruction of tumor vasculature as a result of effective treatment [108,109]. Hypoxia is a major stimulus for angiogenesis, via its stabilization of the hypoxia-inducible factor-1α (HIF-1α) transcription factor [110,111]. HIF-1 is a heterodimeric complex of two helix-loop-helix proteins, HIF-1α and HIF-1β (ARNT). ARNT is constitutively expressed, whereas HIF-1α is rapidly degraded under normoxic conditions. Hypoxia induces the stabilization of the HIF-1α subunit, which, in turn, allows formation of the transcriptionally active protein complex. A number of HIF-1–responsive genes have been identified, including those encoding vascular endothelial growth factor (VEGF), erythropoietin and glucose transporter-1 [112,113]. Following PDT, increases in VEGF secretion and angiogenic responses stimulated via HIF-1 pathways have been documented *in vivo* [114–117]. VEGF induction can contribute to tumor survival and regrowth and, therefore, may represent one of the factors that prevent PDT from achieving its full tumoricidal potential. PDT has been considered for both palliative therapy and as an early treatment option for cancer. Numerous ongoing clinical studies have been designed to optimize PDT conditions. However, no standardized biological markers of cell death and PDT efficacy, other than cell viability itself, have been reported.

**Figure 2:**
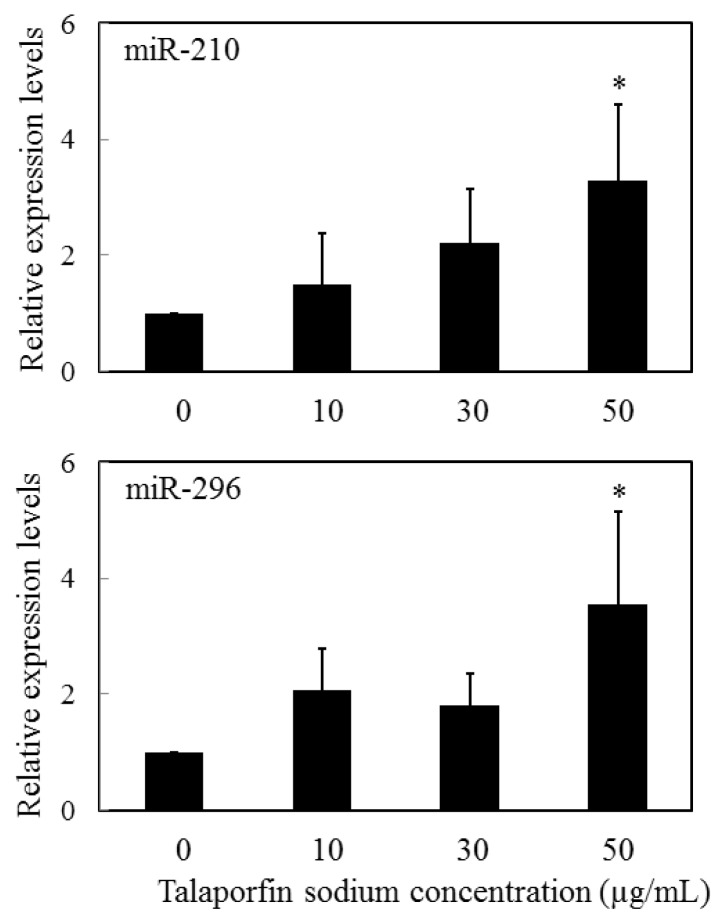
Expression of miR-210 and miR-296 after PDT in HeLa cells. miR-210 and miR-296 expression levels were significantly increased 1 h after PDT (60 mW/cm^2^, 90 s) in cells treated with 50 μg/mL talaporfin sodium relative to levels in the control group (*i.e*., talaporfin sodium concentration of 0 μg/mL) (1 × 10^4^ cells/well). The asterisk, * indicates *p* < 0.05, a significant difference between the relative expression levels of PDT-treated cells and non-PDT-treated cells. All experiments were performed four times independently. All data are expressed as the means ± SD of four replicates from four experiments (Adapted from [120]).

Human cancer is associated with changes in miRNA expression. The pattern of miRNA expression varies dramatically across tumor types, and miRNA profiles reflect the developmental lineage and differentiation state of a tumor [118]. miRNA is also likely to play critical roles in various aspects of hematopoiesis, including the differentiation of hematopoietic stem/progenitor cells, as well as in events that lead to hematological disorders. Nonetheless, very few miRNA expression patterns of specific diseases are available. Moreover, no profiles of miRNA expression after PDT have been reported. Cheng *et al.* found that inhibition of miR-95, -124, -125, -133, -134, -144, -150, -152, -187, -190, -191, -192, -193, -204, -211, -218, -220, -296 and -299 resulted in a decrease in cell growth, whereas inhibition of miR-21 and miR-24 profoundly increased cell growth in HeLa cells [119]. In addition, they identified miRNAs, whose expression increased levels of apoptosis (miR-7, -148, -204, -210, -216 and -296). Those data suggest that specific miRNAs are involved in the cell-death response. We have shown that a miRNA specific to apoptosis is expressed at increased levels in HeLa cells in response to PDT using talaporfin sodium as a photosensitizer [120]. Our study was the first to characterize miRNA expression levels following PDT. In our experiments, miR-210 and miR-296 expression levels increased significantly 1 h after PDT in cells treated with 50 μg/mL talaporfin sodium, relative to the control group (*i.e*., 0 μg/mL talaporfin sodium), as shown in Figure 2. However, the expression levels of other miRNAs, e.g., miR-7, -148a, -204 and -216, were indistinguishable from those of the control group after PDT.

miR-210 is the miRNA most consistently stimulated under hypoxic conditions [121]. Because hypoxia and stabilization of intracellular HIF are inherent consequences of PDT [92], Giannakakis *et al*. investigated miR-210 expression in the context of its hypoxic effect, and they reported evidence for the involvement of the HIF signaling pathway in miR-210 regulation. To study the biological impacts of a partial or complete loss of miR-210 functions, they also identified the putative mRNA targets of miR-210. According to their report, miR-210 targets important regulators of transcription, cell metabolism, differentiation and development, *i.e*., processes that are critically affected by hypoxia [121]. The identification of key regulators of important cellular processes among miR-210 target mRNAs, as well as the high frequency of gene copy-number aberrations in tumors, underscore the involvement of miR-210 in oncogenesis and highlight miR-210 as a potential link between hypoxia and cell-cycle control in cancer cells.

Würdinger *et al*. reported a role for miR-296 in promoting angiogenesis in tumors [122], and in particular, they showed that VEGF alone is capable of increasing miR-296 expression levels. Their results revealed a feedback loop, wherein VEGF induces miR-296 expression, which targets the hepatocyte growth factor-regulated tyrosine kinase substrate (HGS), which, in turn, results in increased levels of VEGF receptor 2 and platelet-derived growth factor (PDGF) receptor β protein and, ultimately, in an increased response to VEGF. Because increased VEGF sensitivity of cancer cells is one of the inherent consequences of PDT [115], our results suggest that inhibition of miR-296 expression should improve PDT efficacy [120]. Our study also suggested that hypoxia induced by PDT induces miR-210 expression, followed by an increased expression of both VEGF and miR-296 [120]. Hence, we reported that miR-210 and miR-296 expression levels represent markers for the efficacy of talaporfin sodium-mediated PDT in cancer cells.

Furthermore, a recently published paper by Bach *et al*. described a comprehensive analysis of changes in miRNA levels following PDT, using polyvinylpyrrolidone hypericin (PVPH) as a photosensitizer, against A431 human epidermoid carcinoma cells [123]. That study was the first comprehensive analysis of changes in miRNA induced by PDT. Using microarray analysis, Bach *et al.* identified eight miRNAs that were significantly differentially expressed 5 hr after treatment, compared with baseline levels, and three miRNAs with more than two-fold differential expression that could be detected in one or two biological replicates. The verification of these results by quantitative real-time PCR, including a detailed time course, revealed an up to 15-fold transient upregulation of miR-634, -1246 and -1290 relative to their basal levels (Table 2).

*In silico* prediction of the targets of these miRNAs yielded numerous mRNAs encoding proteins, including the apoptotic protease activating factor-1 interacting protein and the BMI1 polycomb ring finger oncogene in the apoptosis/cell death category, cyclin-dependent kinase 20 and the cell division cycle 25 homolog C in the proliferation/cell cycle category, frizzled family receptor 3 and bone morphogenetic protein 4 in the cell signaling/adhesion category and the DNA excision repair protein ERCC-8 and peroxiredoxin-6 in the cell stress category. Although several studies have investigated the PDT-induced changes in the transcriptome and proteome, no comprehensive data are currently available regarding the effect of PDT on the miRNA transcriptome. Using a comprehensive microarray platform covering 1223 mature human miRNAs, Bach *et al*. did not observe up- or down-regulation by PDT of the miRNAs reported in our study (miR-210 and -296 [120]). This difference is likely attributable to the PDT conditions, such as cell type, photosensitizer and laser dose. Furthermore, the significant increase in the apoptosis-related miRNAs (3–4-fold increase) observed in our study was measured in a mixed population of cells, consisting predominantly of surviving cells [124]. Given these discrepancies, there is a need for additional experiments that might uncover additional miRNAs that are transiently regulated following photodynamic damage. It will be also of paramount interest to study miRNA-related cellular responses under explicitly non-lethal PDT conditions, as this approach could identify possible miRNA targets, whose manipulation might increase cells’ sensitivity towards PDT.

Interestingly, Bach *et al*. also found that the incubation with the photosensitizer induced a slight to moderate increase in the expression of several miRNAs (*i.e*., miR-1260b, -1260, -1280, -3182, -1290 and -1246), particularly at later time points [123]. Conversely, several miRNAs were transiently up-regulated by light-only treatment, especially at earlier time points (miR-1260b, -1260, -1280, -3182 and -1290). They concluded that the detailed functions of the increased expression of these miRNAs following apoptosis induced by PDT remain to be elucidated [123].

## 3. Conclusions

In this review, we focused on miRNA expression after LLLT and PDT. As mentioned above, only a few papers have been published regarding miRNA expression in this context, and those few reports discuss only a small number of laser therapy conditions. The ability of LLLT to induce growth-factor production, inhibition of inflammation, stimulation of angiogenesis, pain reduction and direct effects on stem cells suggests that there is an urgent need to combine this modality with regenerative medicine. PDT has been employed in the treatment of many tumor types, and its effectiveness as a curative and palliative treatment is well documented, especially in the context of skin cancer. A detailed understanding of LLLT-, phototherapy- and PDT-related molecular mechanisms, including the specific effects on miRNA and protein expression, will provide an important source for new applications of laser therapy and for the development of individualized treatments.

## Figures and Tables

**Table 1:** Aberrations in miRNA expression after low-level laser therapy (LLLT) to mesenchymal stem cells by using a diode laser (wavelength: 635 nm, 0.5 J/cm^2^) [56].

Upregulation	Downregulation
miR-30e ^*^	
miR-15b	
miR-30b-5p	miR-204 ^*^
miR-322	miR-7a
miR-215	miR-423
miR-449a	miR-678
miR-126	miR-25 ^*^
miR-133b	miR-327
miR-21 ^*^	miR-351
miR-455	miR-23a
miR-759	miR-667
miR-872 ^*^	miR-770
miR-29b	miR-324-3p
miR-192	miR-30c-2 ^*^
miR-219-1-3p	miR-758
miR-301a	miR-320
miR-551b	miR-466c
miR-224	
miR-193	

miRNAs expression confirmed by quantitative real-time PCR are indicated by underlining.

The asterisk, ^*^ indicates the star-form of miRNA.

**Table 2:** Aberrations in miRNA expression after PDT to human epidermoid carcinoma cells (A431) by using polyvinylpyrrolidone hypericin (PVPH) [123].

Upregulation	Downregulation
miR-1290	miR-1260b
miR-634	miR-720
miR-1246	miR-1260
	miR-1280
